# Independence in Activities of Daily Living Among Autistic Toddlers: A Pilot Study Using Ecological Momentary Assessment

**DOI:** 10.3390/children12101316

**Published:** 2025-10-01

**Authors:** Shani Hillel, Ben Aaronson, Yafit Gilboa

**Affiliations:** 1 School of Occupational Therapy, Faculty of Medicine, The Hebrew University of Jerusalem, Jerusalem 9124001, Israel; ba1@uw.edu (B.A.); yafit.gilboa@mail.huji.ac.il (Y.G.); 2 Department of Occupational Therapy, Faculty of Social Welfare and Health Sciences, University of Haifa, Haifa 3498838, Israel; 3 Department of Pediatrics, School of Medicine, University of Washington, Seattle, WA 98105, USA

**Keywords:** autism, toddlers, participation, independence, activities of daily living, ecological momentary assessment, parent report

## Abstract

**Background:** The acquisition of adaptive skills is critical for independence and participation in activities of daily living (ADL). While caregiver perceptions provide valuable insights, most studies on autistic participation have focused on older children and relied on one-time clinic-based assessments. As a result, little is known about how autistic toddlers function in their natural environments across time. Ecological momentary assessment (EMA) is a real-time, context-sensitive method in which parents can report behaviors at multiple time points in the child’s natural environment. This pilot study aimed to examine ADL independence in autistic toddlers compared to their typically developing (TD) peers, to assess the feasibility of using EMA in early childhood, and to compare EMA-based assessments with a one-time standardized report. **Methods:** 23 autistic toddlers and 28 TD toddlers (aged 18–40 months) participated in the study. Parents completed a one-time report on the self-care scales of the Pediatric Evaluation of Disability Inventory (PEDI) and the Functional Independence Measure for Children (WeeFIM) and then rated their child’s independence on the WeeFIM twice a day for two weeks via their smartphones. **Results:** EMA was feasible with high response rates (ASD: 91.1%, TD: 88.55%). Autistic toddlers showed different participation profiles, with less independent performance in ADL compared to TD peers. In the autism group, the average EMA scores (*M* = 16.53, *SD* = 6.58) were significantly higher than the one-time WeeFIM scores (*M* = 13.74, *SD* = 5.23), *t* (22) = 3.23, *p* < 0.01, suggesting underreporting in single-time assessments. In contrast, no such difference was found in the TD group. Significant positive correlations were found between the EMA mean and the one-time WeeFIM scores in both groups (*r* > 0.80), indicating convergent validity. In the autism group only, greater variability in EMA was moderately associated with higher functional independence (*r* = 0.46, *p* < 0.01). **Conclusions:** These findings indicate that autistic toddlers demonstrated higher levels of participation in their natural environment than reflected by the one-time assessment, emphasizing the limitations of single-time-point evaluations. This underscores the importance of collecting data across multiple time points to accurately assess adaptive functioning and ADL participation. The EMA technique demonstrated in this study provides exploratory evidence of feasibility as an ecologically valid approach to assessing functional independence in autistic toddlers, offering a richer and more context-sensitive alternative to traditional one-time clinical assessments.

## 1. Introduction

Autism spectrum disorder (ASD) is a lifelong neurodevelopmental condition, characterized by differences in social communication and social interaction across multiple contexts; along with repetitive patterns of behavior, interests, or activities; and distinctive sensory processing [1]. These characteristics are increasingly discussed within a neurodiversity perspective, which emphasizes variability, strengths, and support needs, rather than focusing solely on difficulties [2]. Prevalence has been increasing worldwide [3,4,5]; in the United States, the most recent ADDM surveillance (2022) estimated that about 3.2% (one in 31) of 8-year-old children were identified with ASD in 2022 [6]. Autism is diagnosed more frequently in males than in females, with a male-to-female ratio of 3.4 among 8-year-olds in the U.S. [6], and a median ratio of 4.2 reported in a recent global systematic review [7]. ASD characteristics typically emerge in early childhood, with the earliest observable features appearing at 12–18 months of age [8,9]. Most autistic children can be diagnosed by 18–24 months [3,10].

According to the International Classification of Functioning, Disability and Health (ICF), participation is defined as the individual’s involvement in everyday activities and routines, and is considered a key component of health and functioning [11]. Participation affects children’s behavioral and emotional well-being, social relationships, and overall mental and physical health [12]. Through participation, children develop essential skills, form interpersonal relationships, achieve self-satisfaction, and gain a sense of self-worth [13]. These processes are especially critical during early childhood, a period when foundations for daily occupations such as activities of daily living (ADL) are established [14].

During the toddler years, children are expected to refine adaptive skills, including self-care tasks, and develop independence in activities such as feeding, dressing, and toileting [15]. These years are pivotal for acquiring foundational skills that support future productivity and well-being [10]. ADL in early childhood includes basic self-care activities such as self-feeding, bathing, dressing, personal hygiene, and grooming [16]. Mastery of these skills is supported by concurrent development of fine motor, gross motor, cognitive, and social-emotional abilities [17]. However, due to the unique characteristics of autism, many autistic children and their caregivers factors that may constrain participation opportunities, which can influence both the number and diversity of activities in which they engage [18,19]. Previous studies have shown that nearly 50% of young autistic children are unable to perform self-care activities independently [20], and consistently demonstrate different adaptive skill profiles, characterized by less independent performance in daily living skills relative to their TD peers [21]. These early differences in adaptive skills underscore the need for targeted early intervention to promote independence.

In addition to these challenges, differences in functional activities and adaptive functioning in autistic toddlers can serve as early markers for later developmental outcomes [22]. These differences emphasize the importance of early assessment to monitor development and guide future support and intervention planning. Knowledge of the determinants of self-care participation assists with decisions about how to intervene to support optimal independence [23].

Asking for a caregiver’s perceptions of their child’s ADL participation and competence can provide valuable information about the child’s functioning [24]. However, most previous studies have focused on older children and relied on one-time assessments conducted in clinical settings [20,21]. Thus, little is known about the participation of autistic toddlers in their natural environments over time.

EMA is an intensive longitudinal sampling approach in which parents can report behaviors and contexts at multiple time points, as they occur in their natural environment [25,26]. While EMA methods vary in design, they all share three common characteristics [27]: (1) sampling a behavior or phenomenon over multiple occurrences; (2) observing subjects in their natural environments, increasing the ecological validity of the assessment; and (3) aiming to minimize recall bias by reporting a targeted phenomenon as close to the event as possible. Traditional standardized tools such as the PEDI [28] and WeeFIM [29] are typically administered as one-time reports. However, such parent-report measures may be influenced by parental stress, as parents of children with developmental delays are at heightened risk for chronic stress that can impair memory and comprehension [30], as well as by recall bias and context-specific factors [31]. In contrast, EMA captures multiple instances of behavior across natural settings, providing richer and potentially more valid insights into functional independence, although it does not fully eliminate recall bias [31]. Given the unique aspects of EMA, it has the potential to help bridge knowledge gaps in autism research by capturing real-time experiences that might be missed in traditional assessments [32].

EMA has been employed with the autistic population to report specific areas such as negative emotions and depression [27], social experiences [33,34] and coping [35] as well as to assess leisure participation [36]. However, these studies have focused on adolescent and adult populations. While EMA has been used in these studies to explore emotional and social concerns in autistic individuals, to our knowledge, no EMA study has focused specifically on ADL participation patterns among autistic toddlers.

The main goal of this study was therefore to track the ADL independence profiles of autistic toddlers, and compare them to TD toddlers, using EMA. Our specific aims were to: (a) assess the feasibility of EMA for collecting information regarding ADL among toddlers focusing on a twice-daily measure; (b) describe and compare the independence of the two groups in a variety of ADL; (c) compare the EMA results with standardized one-time assessments to examine their convergent validity; and (d) examine the associations between the standard one-time reports and EMA, in terms of mean and variance.

We hypothesized that: (a) EMA would prove a feasible method for collecting information about ADL participation among the two groups; (b) compared to their TD peers, autistic toddlers would show different participation profiles with less independent involvement exhibit less independent participation in the variety of ADL self-care functions; (c) EMA results would demonstrate convergent validity when compared with standardized one-time measurements; and (d) As an exploratory hypothesis, we examined whether positive correlations would be found between the EMA mean and variability and the one-time report measure.

## 2. Materials and Methods

### 2.1. Participants

The study included a sample of 51 toddlers aged 18–40 months: 23 autistic toddlers and 28 TD toddlers. The sample size was calculated based on a study by Provost et al. [37]. The sample size needed was calculated using G*power version 3.1 and based on a medium-to-large effect size (d = 0.7), a power of 0.8, and α = 0.05., with an allocation ratio of 1:1. The necessary sample size was therefore 52 participants (26 for each group). Using a convenience sampling method, the autistic toddlers were recruited from rehabilitation daycare centers in central Israel, via parent networks and professional contacts, supplemented by a snowball sampling method through media resources. The TD toddlers were recruited through acquaintances and media resources, using snowball sampling. The inclusion criterion for both groups was adequate parental understanding of the Hebrew language.

For the autism group, parents reported that their child had received a clinical diagnosis of autism by either a neurodevelopmental pediatrician, a pediatric neurologist, a child-adolescent psychiatrist, or a psychologist, according to the American Psychiatric Association DSM-5 classification [1], reported by parents. Exclusion criteria were children diagnosed with a developmental disorder other than autism; and/or interfering factors such as significant visual, hearing, or motor deficits. No standardized IQ assessments were administered.

Exclusion criteria for the TD group were any parental concerns in developmental areas of cognitive speech, motor skills, or behavior, according to the child’s parental report. The sample was described by age, gender, number of siblings, parental years of education, and housing density (persons per room) as an indicator of socioeconomic status. These demographic variables were collected to characterize the sample and control for potential confounders in statistical analyses.

### 2.2. Measures

#### 2.2.1. Demographic Questionnaire

An online demographic questionnaire specifically developed for this study included 15 questions to collect personal and socioeconomic information. The questionnaire covered the child’s age at assessment (in months), gender, and number of siblings. Additionally, it collected parental education level and a measure of socioeconomic status, such as housing density (persons per room). Completion time was approximately 5–10 min.

#### 2.2.2. Pediatric Evaluation of the Disability Inventory (PEDI; [28])

PEDI is a functional assessment instrument designed for evaluating function in children with various disabilities aged 6 months to 7.5 years. It is a judgment-based parent-structured interview used by professionals [38]. When completed by a professional, the administrator should have a background in pediatrics, experience with children with disabilities, and strong training in child development [28]. PEDI measures are both functional performance and capability in three domains: (a) self-care, (b) mobility, and (c) social functioning. The functional skills scale consists of 197 items, each scored ‘unable’ (0) or ‘capable’ (1). Administration of the full PEDI (197 items across three domains) typically requires 45–60 min. The final score is the summation of the scoring on all items and can be computed in every domain. In the current study, the Researcher used the self-care domain (73 items) only, which covers eating, grooming, dressing, and personal hygiene, ranging from 0 to 73 [28]. The researcher, a trained occupational therapist, administered the tool in Hebrew, a version that has been previously used [39]. In the present study, completion of the self-care domain typically required 15–20 min. The PEDI has high internal consistency has been found for each of the scales, with Cronbach’s alpha coefficients ranging from 0.95 to 0.99 [28]. PEDI has been suggested as a gold standard measure [19].

#### 2.2.3. Functional Independence Measure for Children (WeeFIM; [29])

WeeFIM is a pediatric functional independence measure developed for children aged 6 months to 7 years with physical and/or mental disabilities. It is administered in a semi-structured interview of around 15 min. The self-care domain used in our study includes eating, grooming, bathing, dressing, and toileting, with each task rated from 1 (requiring total assistance) to 7 (complete independence). The final score sums up all 18 items, ranging from 18 to 126 points. The final score in each category can be referenced to normal scores by age group. The WeeFIM has been widely studied and was found to have strong reliability and validity: internal consistency (Cronbach’s α) is 0.90, interrater-interclass correlation is 0.73–0.94, and test–retest interclass correlation is 0.97 [40].

#### 2.2.4. Ecological Momentary Assessment (EMA)

EMA data reported by the toddlers’ parents provided ecological information about the degree of the child’s dependency in ADL participation. Data was collected through a Qualtrics link sent to the parents’ smartphones twice daily- after the morning routine (8:00 AM) and after the evening routine (8:00 PM), over 10 consecutive weekdays (Sunday–Thursday for two weeks). The EMA form, developed for the study population, comprised all six items of the WeeFIM self-care domain (eating, grooming, bathing, dressing upper body, dressing lower body, and toileting), plus an additional item assessing sleeping/waking as an exploratory item to capture broader daily routines. The introductory instruction read: “Kindly assess the level of independent participation of your child during the current morning/evening in the specified activities” Each item was rated on an 8-point scale: 1 = total assistance; 7 = complete independence; 8 = not relevant. “Not relevant” responses were coded as missing values and excluded from analyses. The meaning of each rating point was explained to parents during the initial session to ensure consistent interpretation. Completion time was approximately 1–3 min.

Two EMA-derived variables were calculated for each participant: (1) mean EMA score, representing the child’s average independence level across all days and activities; and (2) standard deviation of EMA scores, representing variability in independence across contexts and times. Variability was examined to explore whether children whose independence fluctuated more between contexts differed in their one-time assessment scores compared to those with more consistent performance. Final EMA scores were calculated as the average of all items across all responses, and standard deviations were calculated to reflect variability in participation.

### 2.3. Procedure

Approval for the study was obtained from the Ethics Committee for Non-Medical Human Studies in the Faculty of Medicine, The Hebrew University of Jerusalem. Participants provided online informed consent prior to participation. Data collection took approximately 6 months. All interactions with participants were conducted by an occupational therapist. Participants completed an online demographic questionnaire and thereafter, the researcher scheduled a Zoom video meeting with each participant. In this meeting, parents received an explanation of the scales and completed the self-care domains of both the PEDI and the WeeFIM in a semi-structured interview via Zoom, conducted by a licensed occupational therapist with professional training and experience in their administration. In the second stage, every Sunday through Thursday for two consecutive weeks, participants received smartphone messages twice a day, 8 AM and 8 PM, with a link to complete the EMA questionnaire (described in Measures). Thus, each participant contributed up to 20 data points (two prompts per day for 10 days). Parents were encouraged to respond in real time. Response rates and patterns of missing data were recorded to evaluate feasibility and data completeness.

### 2.4. Data Analysis

The variables were analyzed using IBM SPSS software (version 26 Descriptive statistics and normality tests were conducted for all variables. Independent-samples *t* tests and chi-square tests compared the autism and TD groups on demographic variables. Feasibility of the EMA was assessed through calculation of overall and group-specific response rates, as well as examination of missing data patterns across days and time points. EMA means were calculated from available responses only, with missing values (including ‘not relevant’) excluded from the analyses.

Analysis of covariance (ANCOVA) was used to compare groups on the one-time report (WeeFIM), controlling age and maternal education (entered as covariates). Multivariate analysis of covariance (MANCOVA) compared the groups on specific EMA activities, as well as EMA total average and total variability, also controlling for these covariates. Follow-up ANOVAs identified the sources of significant effects. Paired-samples *t* tests compared WeeFIM and EMA scores within groups. Pearson correlation coefficients examined associations between the WeeFIM score and EMA-derived variables (mean and variability). Variability analyses were conducted to explore whether greater fluctuation in daily independence levels was associated with differences in the one-time report, providing insight into contextual effects on ADL performance. Bonferroni corrections were not applied to preserve statistical power, given the pilot nature of the study and the small sample size.

## 3. Results

The demographic characteristics of the two groups are presented in Table 1. Independent *t* tests and chi-square analyses revealed no significant group differences in the number of siblings, father’s years of education, mother’s years of education, or housing density. However, significant differences emerged in both age (*t* = 5.32, *p* < 0.001), with autistic toddlers being older than their TD peers, and gender (*χ*^2^ = 4.31, *p* = 0.03), with a higher proportion of males in the autistic group (78.2%) compared to the TD group (50%). Therefore, age was included as covariate in all subsequent hypothesis-testing analyses. Since no significant gender differences were observed in the dependent variables, gender was not included as a covariate.

*Preliminary Analyses.* Tests of normality (Shapiro–Wilk) were conducted for all dependent variables within each group. Results indicated that most variables did not deviate significantly from normality (all *ps* > 0.05), except for WeeFIM scores in the autistic group (*p* = 0.008).

### 3.1. Aim 1: EMA Feasibilty in the Two Groups

**Figure 1:**
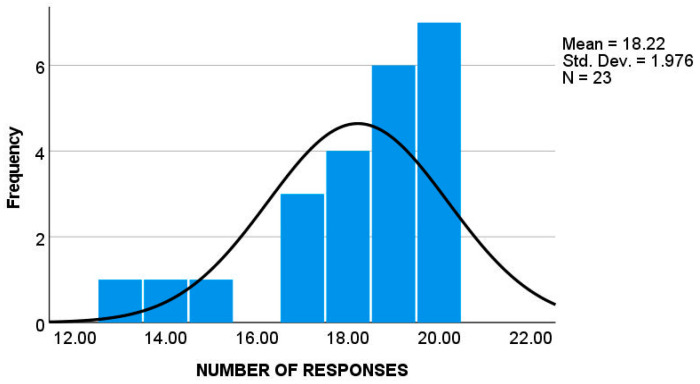
Histogram showing the frequencies of EMA responses in the ASD group.

**Figure 2:**
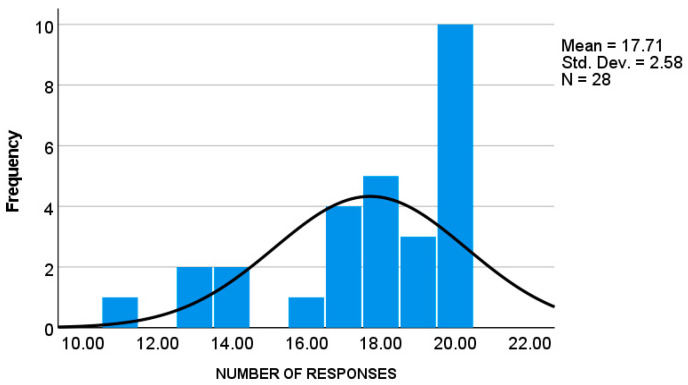
Histogram showing the frequencies of EMA responses in the TD group.

Parents in both groups demonstrated high response rates (autistic group: *M* = 18.22, *SD* = 1.98; *TD* group: *M* = 17.71, *SD* = 2.58), corresponding to 91.1% (Figure 1) and 88.55% (Figure 2) of the maximum possible responses, respectively. An independent *t* test revealed no significant difference between the groups in the number of responses, *t* (49) = 0.76, *p* > 0.5.

### 3.2. Aim 2: Comparison Between Groups of Independence in ADL

The conducted MANCOVA included age and mother education as covariances since they were found to be significantly different between the groups. MANCOVA yielded significant differences between the autistic group and TD groups in PEDI and WeeFIM mean scores *F* (2, 46) = 18.45, *p* < 0.001, *η*^2^ = 0.44. To examine the significance source, each variable’s data were subjected to univariate ANOVA. Significant differences were found in PEDI *F* (1, 47) = 37.53, *p* < 0.001, *η*^2^ = 0.44, and in the WeeFIM *F* (1, 47) = 25.12, *p* = 0.000, *η*^2^ = 0.35. The results of this analysis showed that the TD toddlers received significantly higher scores (indicating better performance) than the children with autism in both one-time report measures. The mean and standard deviations of the measures are presented in Table 2.

To examine whether *Specific activities will differ between the groups*, The MANCOVA applied to the seven EMA variables (eating, grooming, bathing, dressing upper-body, dressing lower-body, toileting, and waking\sleeping) yielded statistically significant differences between the ASD and the TD groups *F* (7, 41) = 3.34, *p* = 0.007, *η*^2^ = 0.36. Univariate ANOVAs were performed on the data for each variable. The mean and standard deviations of the measures are presented in Table 3. The results of this analysis showed that the TD children received significantly higher scores (i.e., performed better) in eating, grooming, bathing, dressing lower-body, toileting, and waking\sleeping variables. No significant group differences were found for upper-body dressing. The mean and standard deviations of the variables were also computed. The average score of the EMA was significantly higher than the TD group, and the variability of the TD group was higher, but it did not reach the significant threshold (*p* = 0.073).

### 3.3. Aim 3: Comparison of One-Time Report Measurment and EMA Results

To enable comparison between the WeeFIM one-time report and the EMA repeated measures, the waking/sleeping activity was excluded from EMA totals. A paired *t* test was conducted, and a significant difference was found only in the autistic group, with the mean score in the one-time report significantly lower than the mean score in the EMA (Table 4).

### 3.4. Aim 4: Relationship Between One-Time Reports and EMA Mean and Variance

Pearson correlation coefficients were calculated separately for each group (Table 5). In both groups, higher EMA mean scores were strongly and significantly associated with higher PEDI and WeeFIM scores, indicating that children who demonstrated greater independence in daily EMA also scored higher in one-time report measures.

**Figure 3:**
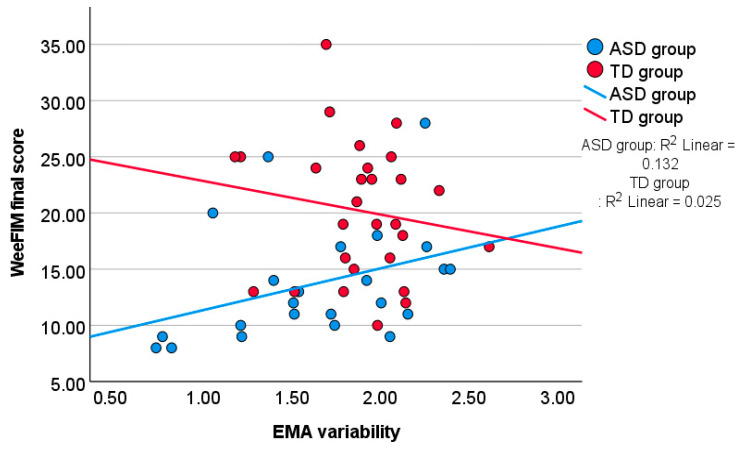
Correlations between the WeeFIM final score and the EMA variability.

In the autistic group, higher EMA variability was significantly associated with higher WeeFIM scores, suggesting that children who showed more fluctuation in independence across contexts also tended to perform better in the WeeFIM. In contrast, in the TD group, correlations between independence and EMA variability were negative but not statistically significant. This difference in trends between groups is illustrated in Figure 3.

## 4. Discussion

The current pilot study aimed to describe independence in ADL performance among autistic toddlers, compared to TD peers, based on parent reports via the EMA approach. EMA was found to be feasible; in fact, the participation rates in both our groups were higher than those reported in other EMA studies conducted among toddlers [26,41]. Previous literature has examined the feasibility of EMA studies using various metrics, with compliance rates being a key indicator. A meta-analysis published in 2023 [42] found that, on average, EMA studies achieved a compliance rate of 79%, though these studies typically included six prompts per day, whereas the present study used only two. In this context, the compliance rates of over 88% in both groups of the study demonstrate strong feasibility. The higher response rate may be attributed to the reminders sent to the participants with the link to the EMA questionnaire twice a day, in line with recommendations for EMA studies [43].

As expected, significant group differences were found in both the one-time reports and the EMA reports, indicating that the autistic group participated less independently in ADL than did TD toddlers. These findings can also be described as reflecting different participation profiles between the groups, rather than solely lower levels of independence. These findings are consistent with previous studies demonstrating that young autistic children show relatively lower performance in self-care tasks, compared to their age norms [21]; and that as a result, autistic children receive more adult support in performing daily self-care activities [44]. Significant differences were found in all activities except upper-body dressing. The absence of group differences in this activity may reflect contextual influences rather than true equivalence in independence. Because data collection occurred during the winter months, even TD toddlers often required parental assistance with layered clothing (e.g., coats, sweaters) [45]. Consequently, upper-body dressing may be less sensitive to developmental differences at this age due to seasonal demands, and findings should therefore be interpreted with caution.

An interesting result of our study was that only parents of autistic toddlers rated their child’s participation significantly lower in their one-time report, compared to the child’s average EMA score. In other words, the everyday ADL functioning of these autistic toddlers was seen to be higher than would be expected from their one-time assessments. One possible explanation for this discrepancy may be parental bias in retrospective reporting. Caregiver ratings are informant-based and not entirely free of bias and may be influenced by caregiver burden. Such biases may lead caregivers to either over- or underestimate the child’s actual functioning [46]. Another possibility is that engagement in the EMA protocol may have gradually raised parents’ awareness of their child’s actual capabilities. Indeed, several parents from the autistic group reported that during the study, they allowed their children to be more independent in a variety of activities. This finding is in line with a previous study that asked caregivers of children with autism to answer daily questions about the child’s irritability, anxiety, and mood, delivered via smartphones across 8 weeks; placebo-like effects were observed, with caregivers reporting symptoms improving over time without explicit treatment [43]. Although promising, this interpretation remains speculative and requires systematic investigation in future work.

In addition, we found that the greater variability in the EMA was associated with higher independence. As suggested by previous studies, the development of self-care functioning among autistic children is characterized by fluctuation and inconsistent performance [47,48]. These fluctuations cannot be measured using one-time reports, whereas it can be documented by EMA. Nevertheless, causality cannot be inferred, and alternative explanations such as inconsistent support, environmental variability, or measurement error should be considered. This association may also reflect methodological differences between retrospective and momentary assessments. Retrospective, one-time measures such as the WeeFIM are susceptible to recall-related biases, as they rely on parents’ memory of past events rather than real-time observations [31]. In contrast, EMA enables the capture of daily fluctuations in naturalistic contexts, as highlighted in studies of youth psychopathology [49], which may help explain the observed correlation between EMA variability and WeeFIM scores in the autistic group. Given the small sample size, the observed association should be considered exploratory until replicated in larger samples.

This study has several limitations. First, the sample size was relatively small, and participants were recruited via convenience and snowball sampling, which may bias toward more motivated or well-connected families, thereby limiting the external validity of the results. Future studies should aim to recruit more representative and diverse samples. Second, autism diagnoses were parent-reported based on professional evaluations but were not independently verified, which reduces diagnostic certainty. Future research should incorporate standardized diagnostic verification procedures to enhance the robustness of findings. Third, the average age of autism diagnosis is typically higher than the average age of the current sample [3], suggesting that the children included may have had higher support needs, as they were identified and diagnosed earlier. Early diagnosis may also be associated with co-occurring lower IQ [50], which could contribute to potential bias. Fourth, no data were collected on participants’ cognitive abilities (IQ) or autism symptom severity, which could have provided further insight into the observed group differences. Further research should consider controlling these factors to improve the generalizability of the findings.

Furthermore, there were significant differences between the groups in some demographic measures. Gender distribution was skewed toward males in the autistic group, consistent with the higher prevalence of autism among boys [6,7]. This imbalance may represent a potential confounder but was not included as a covariate due to the small sample size, which limited statistical power and precluded reliable adjustment for additional variables. Future studies should aim for a more balanced ratio to improve generalizability. Socioeconomic status (SES) was measured only via housing density, a narrow indicator that does not capture other SES dimensions such as income or parental occupation. Future studies should therefore incorporate multiple SES indicators (income, education, occupation) for a more comprehensive assessment. Moreover, weekend routines were not examined despite their potential influence on participation. Finally, multiple analyses were conducted without correction for multiple comparisons to preserve statistical power, given the small sample size and pilot design. This raises the possibility of Type I error, and results should therefore be interpreted with caution. Given the small sample size and absence of correction for multiple comparisons, the significant findings should be regarded as exploratory, providing valuable preliminary evidence that requires confirmation in larger samples. Despite these limitations, this pilot study indicates that EMA may be a feasible approach for assessing daily participation among autistic toddlers and points to avenues for future research.

Beyond the identified limitations, an additional noteworthy finding was that parents reported that their engagement in the study increased their child’s participation, raising the possibility that EMA could serve not only as an assessment tool but also, potentially, as a supportive component within interventions aimed at enhancing daily participation. This observation aligns with the concept of reactivity, defined as the potential for behavior or experience to be affected by the act of assessing it [30]. Although this interpretation should be considered cautiously and examined systematically, it nonetheless suggests an intriguing direction for future research.

## 5. Conclusions

This study used EMA to evaluate independence in ADL among autistic toddlers. Autistic toddlers exhibited different participation profiles, with less independent performance in ADL compared to typically developing peers, particularly in adaptive activities. In the autistic group only, EMA scores indicated higher independence than one-time parent reports, suggesting that EMA may capture a more ecologically valid and dynamic representation of daily functioning. EMA provides exploratory evidence of feasibility as a method for ongoing, ecologically valid assessment in this population. Future studies should build on these findings by recruiting larger and more demographically balanced samples, including weekend routines, integrating parental reports with direct observations, and explicitly monitoring parental reactivity. In addition, future work should adopt neurodiversity-informed perspectives, interpreting differences in participation as reflecting diverse developmental trajectories and support needs.

## Figures and Tables

**Table 1:** Demographic characteristics of the two groups.

Demographic Information	Autistic Group (*n* = 23)	TD Group (*n* = 28)	
Age at assessment, months; *M* (*SD*), range	32.13 (4.65), 23–40	24.93 (4.86), 18–35	*t* = 5.32 (*p* < 0.001)
Gender (% males)	18 (78.2)	14 (50)	*χ*^2^ = 4.31 (*p* = 0.03)
Number of siblings; *M*	0.87	1.07	*t* = −0.68 (*p* = 0.50)
SES			
Mother years of education; *M* (*SD*), range	15.04 (2.55), 12–20	16.71 (2.17), 15–22	*t* = −2.52 (*p* = 0.15)
Father years of education; *M* (*SD*), range	14.52 (2.27), 12–18	14.71 (3.65), 8–22	*t* = −0.22 (*p* = 0.83)
Housing density, *M* (*SD*)	0.94 (0.24)	1.06 (0.34)	*t* = −1.35 (*p* = 0.18)

*Note.* SES = Socioeconomic Status; Housing density—persons per room. M = mean, SD = Standard deviation.

**Table 2:** Comparison of the PEDI and the WeeFIM Measures Between the Groups.

	Autistic Group (*n* = 23)		TD Group (*n* = 28)			
	*M*	*SD*	*M*	*SD*	*F*	*η* ^2^
PEDI	25.61	12.44	41.43	9.63	16.02 ***	0.44
WeeFIM	13.74	5.24	20.21	6.01	12.93 ***	0.35

*Note.* PEDI = Pediatric Evaluation of Disability Inventory; WeeFIM = The Functional Independence Measure for Children. *** *p* < 0.001.

**Table 3:** Comparison of EMA results between the two Groups.

	Autistic Group (*n* = 23)		TD Group (*n* = 28)			
	*M*	*SD*	*M*	*SD*	*F*	*η* ^2^
Eating	5.03	1.69	6.22	0.68	4.69 **	0.23
Grooming	2.45	1.37	3.04	1.37	5.23 **	0.25
Bathing	2.18	1.19	3.08	1.35	7.62 ***	0.33
Dressing-Upper Body	2.42	1.17	2.63	0.97	2.32	0.13
Dressing-Lower Body	2.33	1.07	3.22	1.51	7.54 ***	0.32
Toileting	2.12	1.65	2.19	1.91	6.03 ***	0.28
Waking\Sleeping	4.27	1.97	5.54	1.55	4.12 **	0.21
Total average	2.97	1.12	3.70	0.98	8.42 ***	0.35
Total variability	1.64	0.51	1.88	0.32	2.47	0.14

*Note.* EMA-Ecological Momentary Assessment. ** *p* < 0.01. *** *p* < 0.001.

**Table 4:** Comparison between the WeeFIM and the EMA results within the groups.

	WeeFIM		EMA		
	*M*	*SD*	*M*	*SD*	*t*
Autistic group	13.74	5.23	16.53	6.58	3.23 **
TD group	20.21	6.01	20.40	6.04	0.3

*Note.* WeeFIM = The Functional Independence Measure for Children; EMA-Ecological Momentary Assessment. ** *p* < 0.01.

**Table 5:** Correlations coefficients of one-time report measures and EMA mean and variability.

		EMA Mean	EMA Variability
Autistic group	PEDI	0.76 **	0.4
	WeeFIM	0.84 **	0.46 **
TD group	PEDI	0.79 **	−0.12
	WeeFIM	0.88 **	−0.22

*Note.* PEDI = Pediatric Evaluation of Disability Inventory; WeeFIM = The Functional Independence Measure for children. ** *p* < 0.01.

## Data Availability

The datasets generated and analyzed during the current study are not publicly available due to ethical and privacy restrictions but are available from the corresponding author upon reasonable request.

## Abbreviations

The following abbreviations are used in this manuscript:

Abbreviation	Definition
ASD	Autism Spectrum Disorder
DSM	Diagnostic and Statistical Manual of Mental Disorders
ADL	Activities of Daily Living
PEDI	Pediatric Evaluation of Disability Inventory
WeeFIM	Functional Independence Measure for Children
EMA	Ecological Momentary Assessment
TD	Typically Developing
SES	Socioeconomic Status
G*Power	G*Power (Statistical Power Analysis Software)
